# Physical Performance Moderates the Association Between Age and Executive Function in Older Adults at Elevated Risk of Dementia: Cross‐Sectional Analysis From the CITA GO‐ON Trial

**DOI:** 10.1002/jcsm.70396

**Published:** 2026-10-05

**Authors:** Imanol Reparaz‐Escudero, Mikel Izquierdo, Mirian Ecay‐Torres, Miren Altuna, Carolina López, Ainara Estanga, Maite García‐Sebastián, María de Arriba, Naia Ros, Jon Saldias, Marina Limousin, Pablo Martínez‐Lage, Mikel L. Sáez de Asteasu, The CITA GO‐ON Study Group

**Affiliations:** ^1^ Navarrabiomed, Hospital Universitario de Navarra (HUN)‐Universidad Pública de Navarra (UPNA), IdiSNA Pamplona Spain; ^2^ Centre for Research and Memory Clinic CITA‐Alzheimer Foundation Donostia‐San Sebastián País Vasco Spain; ^3^ CIBER of Frailty and Healthy Aging (CIBERFES), Instituto de Salud Carlos III Madrid Spain; ^4^ Department of Neurology, Bioaraba Health Research Institute Araba University Hospital‐Txagorritxu Vitoria‐Gasteiz Spain

**Keywords:** dementia, functional capacity, physical performance

## Abstract

**Background:**

Preservation of executive function is vital for independence and dementia prevention, yet the physical performance levels at which age‐related executive vulnerability becomes detectable remain uncertain. This study examined whether gait speed, handgrip strength and leg muscle power influence the relationship between age and executive function in older adults at increased dementia risk and assessed how sex and white matter hyperintensity (WMH) burden affect this relationship.

**Methods:**

Cross‐sectional analysis of 920 community‐dwelling older adults (aged 60–85) at high dementia risk, enrolled in the CITA GO‐ON trial. Executive function was measured by the Trail Making Test (TMT B minus A) and the Stroop interference score. Physical performance included gait speed, handgrip strength and peak leg muscle power. Moderation analyses tested whether physical performance moderated the age–executive function association, adjusting for sex, socioeconomic status, depressive symptoms, cardiometabolic conditions, BMI and smoking; three‐way interactions tested sex effects. The Johnson–Neyman technique identified regions of significance, the range of performance values over which the age–executive function association was statistically detectable. Sensitivity analyses included cognitively healthy participants and WMH adjustment.

**Results:**

The average (SD) age was 69.5 (6.2) years; 48.4% were female, and average education was 13.0 (4.5) years. At baseline, 63 participants (6.8%) had mild cognitive impairment. Gait speed (*β* = −0.27, *p* < 0.05), handgrip strength (*β* = −0.08, *p* < 0.05) and peak leg muscle power (*β* = −0.007, *p* < 0.05) moderated the age–executive function link. Johnson–Neyman regions‐of‐significance were located in the upper range of the performance distribution. For TMT B minus A, the age–executive function association was statistically detectable below gait speed 1.83 m/s, handgrip 48.8 kg and leg power 438 W (1.0%–4.8% of participants), whereas for Stroop interference the corresponding values were lower (gait 1.68 m/s, handgrip 43.9 kg, leg power 366 W; up to 15.9% of participants). In sex‐stratified analyses, gait speed moderated the age–executive function association for TMT B minus A in males and for Stroop interference in females. WMH burden was not independently associated with executive function.

**Conclusions:**

In older adults at increased dementia risk, higher physical performance was associated with a weaker relationship between age and executive function, independent of socioeconomic, emotional, body composition and cardiovascular factors. This moderation was most consistent for gait speed, with a sex pattern that differed by outcome. Longitudinal studies are needed to test whether maintaining high physical performance helps preserve executive function and inform preventive strategies.

## 1 Introduction

Dementia, characterized by progressive cognitive decline and loss of independence, has been identified as a major public health challenge of our era [1]. Predictions indicate an unprecedented global increase in the number of people affected by dementia, rising from 50 million in 2020 to nearly 150 million by 2050 [2, 3]. The most common causes are Alzheimer's disease (AD) and vascular dementia, responsible for 60%–70% and 10%–20% of cases, respectively. However, in 30%–40% of cases, AD and vascular pathology coexist, shaping cognitive decline trajectories together (e.g., mixed dementia) [4, 5, 6]. Despite promising advances in pharmacological treatments for early Alzheimer's disease [7, 8], current disease‐modifying therapies are insufficient to curb the dementia epidemic at the population level. Recent intergenerational post‐mortem studies have shown no difference in neuropathological burden across birth cohorts, suggesting that improvements in cerebrovascular health and factors promoting cognitive reserve may explain the observed reductions in age‐adjusted dementia incidence [9]. The 2024 Lancet Commission on Dementia Prevention, Intervention, and Care reported that up to 45% of dementia cases could be preventable through modification of 14 risk factors, including early‐life factors (e.g., low education), midlife factors (e.g., hypertension, obesity, physical inactivity, hearing loss and high LDL cholesterol) and late‐life factors (e.g., social isolation, air pollution and vision loss) [10]. Collectively, these findings highlight the importance of preventive strategies targeting vascular health, metabolic health and cognitive reserve as essential components of public health efforts to prevent dementia.

Among the cognitive domains affected by aging, executive functions are especially vulnerable [11]. Early impairments in planning, inhibition and cognitive flexibility disrupt instrumental activities of daily living (IADLs) [12, 13], decrease social participation, negatively affect quality of life [10] and ultimately raise the risk of dementia [14, 15]. Physical inactivity is a major midlife factor contributing to dementia, leading to increases in obesity, diabetes, dyslipidaemia, hypertension and cardiometabolic disease [16] and also influencing depression [17]. Beyond these systemic effects, physical activity levels are consistently linked to executive function over time [18], further influencing the long‐term risk of dementia [19, 20, 21]. Objective markers of physical performance, such as gait speed, handgrip strength and muscle power, offer valuable insights into long‐term behavioural patterns. These measures are associated with vascular metabolic health and functional resilience and predict independence and the development of dementia in older adults [22, 23, 24, 25, 26]. In fact, these measures may serve as early clinical indicators of cognitive vulnerability, detectable years before dementia onset [27].

From a muscle‐centric perspective, gait speed, handgrip strength and leg muscle power can be viewed as integrative phenotypes reflecting multisystem reserve—neuromuscular capacity, cardiometabolic health and central motor control—rather than isolated fitness traits. These markers are strongly prognostic for disability and mortality and are increasingly linked to cognitive trajectories, suggesting that superior physical performance may buffer the expression of brain aging. Testing physical performance as a moderator of age‐related executive vulnerability therefore offers a complementary lens to conventional main‐effect associations, aligned with precision‐prevention approaches within WW‐FINGERS‐type multidomain programmes.

Cerebrovascular pathology burden is also a key factor in cognitive aging [28], linked to faster decline in executive function among older adults [29, 30, 31]. White matter hyperintensities (WMHs) are a hallmark of cerebral small vessel disease and indicate accumulated ischemic damage to microvascular structures in the brain. High WMH loads predict a greater risk of cerebrovascular events, cognitive impairment and dementia [32, 33, 34]. Because of its central role in shaping executive functions, studying the impact of WMH burden is essential to understanding cognitive resilience related to physical performance.

While the primary connections between physical performance and executive function have been widely shown, no previous research has specifically looked at physical function as a moderator of the harmful effects of aging on executive function. Similarly, no study has characterized the range of physical performance values under which this moderation is statistically detectable, which could inform the design of personalized physical exercise and lifestyle strategies.

Therefore, we examined whether gait speed, handgrip strength and leg muscle power influence the relationship between age and executive function in community‐dwelling older adults at risk for dementia. Additionally, we used Johnson–Neyman (JN) analyses to delineate the regions‐of‐significance of physical performance for this moderation and investigated the potential roles of sex and cerebral WMH burden.

## 2 Methods

### 2.1 Design

Cross‐sectional analysis of baseline participant data from the CITA GO‐ON study (NCT04840030), an ongoing FINGER‐like, community‐based randomized controlled trial assessing the effect of a multidomain nonpharmacological intervention on preventing cognitive decline and dementia. The study was conducted at the CITA‐Alzheimer Foundation in the Basque Country, Spain. The detailed trial protocol is available online [35]. Ethical approval was granted by the Euskadi Drug Research Ethics Committee (ID: PI2020244), and all participants provided written informed consent to take part in the study.

### 2.2 Participants and Settings

Data were collected between July 2021 and February 2024 at the CITA Alzheimer Foundation, located in Donostia/San Sebastián. The detailed process of participant screening and recruitment has been previously published [35]. Briefly, the community‐based recruitment strategy included collaborating with local town halls, issuing press releases, running social media campaigns, conducting large‐scale direct mailings and hosting community talks. Interested individuals completed an online prescreening RedCap questionnaire that included basic sociodemographic information and self‐reported data relevant to the study's selection criteria. To be eligible for inclusion, participants had to be 60–85 years old, at high risk of dementia based on the Cardiovascular Aging and Dementia risk score (CAIDE ≥ 6) and show lower‐than‐expected cognitive performance on one out of two brief cognitive tests (≤ 35 points in the Fototest score [36] or ≤ 40 points on the Memory Alteration Test score [37] or have subjective cognitive decline [SCD; ≤ 20 points on the first 12 items of the Cognitive Change Index]). Patients diagnosed with dementia or who had lost functional independence (Barthel Index < 90 points) were excluded. If participants met the eligibility criteria, they were invited to attend an in‐person screening appointment to confirm their eligibility and provide informed consent.

A total of 920 participants were included in this study (Figure S1). For the main analysis, we included participants with baseline data on the outcomes of interest (e.g., TMT‐B minus TMT‐A or Stroop interference score), the main predictor (baseline age), covariates and each examined moderator (gait speed, handgrip strength and peak leg muscle power).

### 2.3 Data Collection

Data were collected in a computerized database using an electronic clinical record form (eCRF) record system based on REDCap [38, 39]. REDCap is a secure web‐based software platform designed to support data capture in research studies.

### 2.4 Measurements

#### 2.4.1 Sociodemographic, Clinical and Anthropometric Characteristics

The sociodemographic and clinical characteristics collected at baseline included age, sex, educational level (years of education), presence and extent of depressive symptoms [40] (Geriatric Depression Scale [GDS]) and body mass index (BMI). The Hollingshead Four‐Factor Index [41] was used to measure socioeconomic status (SES) based on formal education and professional categories. The total score ranges from 11 to 77, with scores of 11–17 indicating a high position, 18–31 indicating a medium‐high position, 32–47 indicating a middle position, 48–63 indicating a medium‐low position and 64–77 indicating a low position. For analysis and reporting, this score was recoded into a five‐level ordinal index ranging from 0 (lowest position, Hollingshead 64–77) to 4 (highest position, Hollingshead 11–17), so that higher values denote higher socioeconomic status. In addition to education, factors related to job requirements and household income have been shown to positively affect cognitive reserve, independent of education alone [42]. Moreover, higher SES allows access to healthcare resources and healthier lifestyle choices, thereby promoting cognitive reserve and brain health. MCI diagnosis was confirmed at the medical prerandomization visit by study neurologists after a comprehensive neuropsychological assessment, following Petersen criteria [43].

#### 2.4.2 Executive Function

The primary outcome for executive function was calculated as the difference between the completion times of the Trail Making Test Part A (TMT‐A) and Part B (TMT‐B). The Trail Making Test (TMT) is a well‐established tool for assessing executive functioning. In the first part of the test (TMT‐A), participants are instructed to manually connect circles with numbers in sequential order (1–2–3, etc.), recording the completion time and the number of errors. The second part (TMT‐B) follows a similar format but involves alternating numbers and letters. Participants are required to connect numbers and letters in an interleaved sequence (1–A–2–B–3–C, etc.), which increases cognitive demands. The difference score (TMT‐B minus TMT‐A) reduces the influence of baseline motor speed, visuoperceptual ability and visual scanning, thereby providing a purer, though not exclusive, index of executive demands such as cognitive flexibility and task‐switching [44]. Lower scores indicate better executive function.

The Stroop Colour and Word Test was included as a co‐primary outcome. The following three conditions were presented in this test: reading colour words printed in black (word–colour), naming the colours of coloured rectangles (colour–naming), and the interference condition, where colour words are printed in incongruent colours and participants must name the ink colour while ignoring the word (colour–naming). Participants' performance was recorded as the completion time and the number of errors in each condition. For this study, the Stroop interference score was calculated as the difference between the time taken to complete the incongruent word–colour condition and the colour–naming condition. Lower scores indicated better executive function.

#### 2.4.3 Physical Performance

Usual gait speed, handgrip strength and leg muscle power were used to assess physical performance. Usual gait speed was measured over a 6‐m walk along a flat, indoor corridor. Two trials were performed at the participants' self‐selected usual walking pace, and the better of the two trials was used to calculate walking speed (meters per second, m/s). Handgrip strength (kg) was measured with a handheld dynamometer (T.K.K. 5401 Grip‐D, Japan). The participants stood upright with their dominant arm fully extended and slightly abducted. The study physiotherapist provided standardized verbal instructions to encourage participants to squeeze as hard as possible, and the highest of two maximal efforts lasting 3–4 s was recorded. Lower limb peak muscle power (W) was assessed using a linear encoder (Vitruve, VBT, Spain) attached to the leg press machine. After a progressive one‐repetition maximum (1RM) test, participants performed 10 repetitions at 50% of their 1RM, aiming for maximum concentric velocity. The highest peak power value (W) was then recorded.

#### 2.4.4 MRI Derived WMH Burden

The MRI‐derived Fazekas scale [45] was used to assess the burden of WMH. The Fazekas scale is a semiquantitative visual scale that grades WMH in the periventricular and deep subcortical regions on a 0–3 scale, in ascending severity. Participants underwent 3T MRI following a standardized protocol for obtaining axial FLAIR images that were subsequently evaluated by the study neurologists based on the Fazekas scale.

### 2.5 Statistical Analysis

Data are expressed as mean ± standard deviation (SD) for continuous variables and absolute numbers and percentages for categorical variables. Independent *t*‐tests or chi‐square tests were applied to examine baseline sex differences.

Three multiple linear regression models were fitted per executive outcome, with baseline age (in years) as the primary predictor variable. Sex, SES, depressive symptoms, BMI, anxiety, diabetes, hypertension, dyslipidaemia and smoking status were included as covariates. Each model included a two‐way interaction term between age and physical performance (gait speed, handgrip strength or leg muscle power) to test whether physical performance moderated the association between age and executive function (i.e., TMT‐B minus TMT‐A and Stroop interference score). Because two related executive outcomes were examined across three physical‐performance markers, the moderation hypothesis was considered supported if either showed a significant age × performance interaction. We applied a Benjamini–Hochberg false‐discovery‐rate (FDR) correction to the six primary age × performance interactions to guard against type I error inflation. To ensure that main‐effect coefficients were interpretable within the observed data range, age and all physical performance markers were mean‐centred prior to fitting the interaction models. Gait speed was rescaled to 0.1 m/s units, so that its coefficients reflect the change in executive performance per 0.1 m/s increment. In addition to raw‐unit coefficients, standardized coefficients were computed.

Models with a three‐way interaction term (age × sex × physical function) were built to evaluate the specific influence of sex on the association. Finally, we explored the potential influence of WMH on executive functioning.

When significant moderation effects were detected, the JN technique was applied to delineate the regions‐of‐significance, that is, the range of physical performance values above which age was not significantly associated with executive function [46]. Multiple linear regression models and JN analyses were stratified by sex.

All analyses were conducted using R Version 4.4.3. Multiple linear regression models were fitted using the lm() function. The JN technique was implemented using the *interactions* package (v. 1.2.0). A two‐sided *α* level of 0.05 was used.

### 2.6 Sensitivity Analyses

First, to assess the robustness of our findings, we replicated the primary models in a subset of cognitively unimpaired participants (CU) to reduce the influence of participants diagnosed with MCI. Second, we re‐estimated the primary moderation models including sleep quality (Pittsburgh Sleep Quality Index total score) as a further covariate. Because of missing PSQI data, this variable was not included in the primary models to preserve sample size. Third, to address the body size dependence of grip strength and leg power, we re‐estimated the moderation models using relative grip strength (kg/BMI) and relative leg power (W/kg), without BMI as a covariate. Similarly, gait speed models were re‐estimated adjusting for standing height.

## 3 Results

### 3.1 Participants

The study flow diagram is shown in Figure S1. Table 1 displays the characteristics of the study participants, including sex‐specific comparisons of sociodemographic, clinical, cognitive and physical performance data. A total of 920 older adults were included, with a mean (SD) age of 69.5 (6.1) years and 48.4% women. Participants had an average of 13.0 (4.5) years of formal education, and 63 (6.8%) met the diagnostic criteria for MCI at baseline. The level of depressive symptoms was low, with a mean GDS score of 1.94 (2.2) points. The burden of white matter hyperintensities (WMH) was moderate to high (Fazekas score ≥ 2) in 28.7% of participants, indicating a significant presence of cerebrovascular pathology.

**TABLE 1:** Descriptive sociodemographic, clinical, cognitive and physical characteristics of study participants, with sex‐wise group comparisons.

Characteristic	*n*	Overall *N* = 920	Female *n* = 445	Male *n* = 475	Difference	95% CI	*p*
Age, years	920	69.55 (6.19)	69.94 (6.48)	69.19 (5.88)	0.75	−0.06, 1.6	0.068
Education level, years	920	13.00 (4.48)	12.13 (4.48)	13.82 (4.33)	−1.7	−2.3, −1.1	< 0.001***
Socioeconomic status index	920				−0.56	−0.69, −0.43	
0		163 (18%)	128 (29%)	35 (7.4%)			
1		159 (17%)	73 (16%)	86 (18%)			
2		280 (30%)	130 (29%)	150 (32%)			
3		303 (33%)	112 (25%)	191 (40%)			
4		15 (1.6%)	2 (0.4%)	13 (2.7%)			
Syndromic diagnosis	920				0.13	0.00, 0.26	
Cognitively healthy		806 (88%)	380 (85%)	426 (90%)			
Subjective cognitive decline		51 (5.5%)	28 (6.3%)	23 (4.8%)			
Mild cognitive impairment		63 (6.8%)	37 (8.3%)	26 (5.5%)			
Depression, GDS score	919	1.94 (2.16)	2.28 (2.34)	1.62 (1.93)	0.65	0.37, 0.93	< 0.001***
Body mass index	917	27.41 (4.70)	27.00 (5.23)	27.79 (4.11)	−0.79	−1.4, −0.18	< 0.05*
Fazekas score, 0–3	774				0.16	0.02, 0.30	
0		166 (21%)	67 (18%)	99 (25%)			
1		381 (49%)	187 (50%)	194 (49%)			
2		197 (25%)	105 (28%)	92 (23%)			
3		30 (3.9%)	15 (4.0%)	15 (3.8%)			
TMT‐B minus TMT‐A, time in seconds	841	63.81 (55.52)	69.42 (61.85)	58.89 (48.83)	11	2.9, 18	< 0.01**
Stroop interference, time in seconds	907	27.05 (15.42)	28.66 (17.13)	25.51 (13.42)	3.2	1.1, 5.2	< 0.01**
Usual gait speed 6 m, m/s	898	1.32 (0.23)	1.28 (0.22)	1.35 (0.24)	−0.06	−0.09, −0.03	< 0.001***
Handgrip, kg	893	29.21 (8.83)	21.97 (3.87)	35.97 (6.47)	−14	−15, −13	< 0.001***
Peak leg muscle power, W	863	243.54 (105.69)	166.67 (52.24)	314.74 (91.92)	−148	−158, −138	< 0.001***

*Note:* Mean (standard deviation); *n* (%). 79 participants were unable to complete the TMT‐B task due to alphabetical illiteracy. Additionally, 22 (2.4%) observations on gait speed, 27 (2.9%) on handgrip strength, and 58 (6.3%) on peak leg muscle power were missing owing to absolute or relative contraindications, unwillingness to perform the established evaluations or staff shortage.

Abbreviations: 95% CI, 95% confidence intervals; BMI, body mass index; GDS, Geriatric Depression Scale; TMT, Trail Making Test.

* *p* < 0.05.

** *p* < 0.01.

*** *p* < 0.001.

### 3.2 Sex Differences

Marked sex differences were observed in the sociodemographic characteristics. Females had lower educational attainment (mean difference [MD] 1.7 years, *p* < 0.001), were overrepresented in the lowest SES categories (29.0% vs. 7.4%) and were underrepresented in the highest SES categories (0.4% vs. 2.7%). Females also had slightly higher depression scores (MD 0.65, *p* < 0.001) and lower BMI (MD 0.8, *p* < 0.05).

Males outperformed females in physical performance measures, with significantly higher gait speed (MD 0.06 m/s, *p* < 0.001), handgrip strength (MD 14 kg, *p* < 0.001) and peak leg muscle power (MD 158 W, *p* < 0.001) than females. In cognitive outcomes, females performed worse on the TMT B minus A (MD ~ 11 s, *p* < 0.01) and the Stroop interference task (MD 3.2 s, *p* < 0.01). No significant sex differences were observed in the Fazekas scores.

### 3.3 Moderation Analyses

#### 3.3.1 TMT B Minus A

In the fully adjusted models, older age and greater depressive symptom burden were consistently associated with poorer executive functioning, whereas higher SES (indexed by education, occupational complexity and income) exerted a robust protective effect (Table 2).

**TABLE 2:** Multiple regression of sociodemographic and clinical predictors on executive function. Models are adjusted for the full covariate set and the age × performance interaction.

Outcome	Predictor	Performance model	Whole *β* (95% CI)	*p*	Female *β* (95% CI)	*p*	Male *β* (95% CI)	*p*
TMT B minus A	Age (years)	Gait speed	2.88 (2.25 to 3.51)	< 0.001***	3.26 (2.22 to 4.31)	< 0.001***	2.55 (1.76 to 3.34)	< 0.001***
		Handgrip	2.97 (2.38 to 3.56)	< 0.001***	3.28 (1.55 to 5.01)	< 0.001***	3.39 (2.29 to 4.49)	< 0.001***
		Leg muscle power	2.64 (2.04 to 3.24)	< 0.001***	2.62 (1.06 to 4.18)	< 0.01**	2.43 (1.54 to 3.33)	< 0.001***
	Sex (ref. female)	Gait speed	2.46 (−4.86 to 9.78)	0.51	—	—	—	—
		Handgrip	5.91 (−5.38 to 17.21)	0.3	—	—	—	—
		Leg muscle power	7.52 (−2.14 to 17.17)	0.13	—	—	—	—
	Socioeconomic status index	Gait speed	−13.53 (−16.89 to −10.17)	< 0.001***	−14.55 (−19.58 to −9.51)	< 0.001***	−10.85 (−15.50 to −6.20)	< 0.001***
		Handgrip	−12.91 (−16.13 to −9.69)	< 0.001***	−13.31 (−17.86 to −8.76)	< 0.001***	−11.71 (−16.45 to −6.97)	< 0.001***
		Leg muscle power	−13.35 (−16.49 to −10.21)	< 0.001***	−13.26 (−17.92 to −8.59)	< 0.001***	−12.31 (−16.72 to −7.90)	< 0.001***
	Depression (GDS)	Gait speed	1.90 (0.14 to 3.66)	< 0.05*	0.99 (−1.65 to 3.64)	0.46	3.29 (0.86 to 5.72)	< 0.01**
		Handgrip	2.10 (0.44 to 3.76)	< 0.05*	1.18 (−1.19 to 3.55)	0.33	3.37 (0.91 to 5.84)	< 0.01**
		Leg muscle power	2.05 (0.43 to 3.67)	< 0.05*	1.01 (−1.40 to 3.43)	0.41	3.68 (1.40 to 5.96)	< 0.01**
	Anxiety	Gait speed	0.57 (−1.00 to 2.15)	0.47	0.83 (−1.56 to 3.21)	0.5	0.25 (−1.90 to 2.39)	0.82
		Handgrip	0.96 (−0.53 to 2.46)	0.21	1.40 (−0.77 to 3.56)	0.2	0.50 (−1.68 to 2.68)	0.65
		leg muscle power	1.01 (−0.45 to 2.47)	0.18	1.47 (−0.73 to 3.67)	0.19	0.28 (−1.75 to 2.31)	0.79
	BMI	Gait speed	−0.66 (−1.44 to 0.12)	0.1	−0.83 (−1.98 to 0.32)	0.16	−0.49 (−1.61 to 0.63)	0.39
		Handgrip	−0.39 (−1.12 to 0.33)	0.29	−0.42 (−1.39 to 0.55)	0.39	−0.40 (−1.55 to 0.74)	0.49
		Leg muscle power	−0.21 (−0.94 to 0.53)	0.58	−0.15 (−1.19 to 0.89)	0.78	−0.24 (−1.35 to 0.87)	0.67
Stroop interference	Age (years)	Gait speed	0.53 (0.38 to 0.69)	< 0.001***	0.58 (0.34 to 0.82)	< 0.001***	0.44 (0.23 to 0.66)	< 0.001***
		Handgrip	0.59 (0.44 to 0.75)	< 0.001***	0.57 (0.13 to 1.00)	< 0.05*	0.60 (0.30 to 0.90)	< 0.001***
		Leg muscle power	0.47 (0.30 to 0.64)	< 0.001***	0.42 (0.01 to 0.83)	< 0.05*	0.47 (0.21 to 0.73)	< 0.001***
	Sex (ref. female)	Gait speed	0.74 (−1.10 to 2.58)	0.43	—	—	—	—
		Handgrip	2.12 (−0.93 to 5.16)	0.17	—	—	—	—
		Leg muscle power	4.11 (1.44 to 6.78)	< 0.01**	—	—	—	—
	Socioeconomic status index	Gait speed	−3.30 (−4.12 to −2.48)	< 0.001***	−3.07 (−4.23 to −1.90)	< 0.001***	−3.31 (−4.52 to −2.09)	< 0.001***
		Handgrip	−3.35 (−4.19 to −2.52)	< 0.001***	−3.08 (−4.25 to −1.90)	< 0.001***	−3.40 (−4.63 to −2.18)	< 0.001***
		Leg muscle power	−3.26 (−4.11 to −2.42)	< 0.001***	−3.00 (−4.21 to −1.78)	< 0.001***	−3.21 (−4.43 to −1.99)	< 0.001***
	Depression (GDS)	Gait speed	0.50 (0.06 to 0.93)	< 0.05*	0.45 (−0.16 to 1.06)	0.15	0.64 (−0.02 to 1.30)	0.06
		Handgrip	0.55 (0.11 to 0.99)	< 0.05*	0.47 (−0.14 to 1.08)	0.13	0.70 (0.04 to 1.36)	< 0.05*
		Leg muscle power	0.58 (0.14 to 1.03)	< 0.05*	0.46 (−0.16 to 1.08)	0.15	0.83 (0.16 to 1.49)	< 0.05*
	Anxiety	Gait speed	−0.04 (−0.43 to 0.35)	0.84	0.13 (−0.41 to 0.67)	0.64	−0.30 (−0.88 to 0.28)	0.31
		Handgrip	−0.01 (−0.40 to 0.38)	0.95	0.12 (−0.43 to 0.66)	0.67	−0.26 (−0.85 to 0.33)	0.39
		Leg muscle power	0.01 (−0.38 to 0.41)	0.95	0.16 (−0.39 to 0.71)	0.56	−0.27 (−0.86 to 0.32)	0.36
	BMI	Gait speed	−0.13 (−0.33 to 0.07)	0.21	−0.16 (−0.43 to 0.11)	0.23	−0.13 (−0.44 to 0.18)	0.43
		Handgrip	−0.05 (−0.24 to 0.15)	0.63	−0.05 (−0.30 to 0.21)	0.71	−0.08 (−0.39 to 0.24)	0.63
		Leg muscle power	−0.01 (−0.22 to 0.19)	0.91	0.02 (−0.26 to 0.29)	0.91	−0.06 (−0.39 to 0.27)	0.74

*Note:* Models are additionally adjusted for diabetes, hypertension, dyslipidaemia and smoking (not shown) and show centred, raw‐unit betas. Gait speed expressed per 0.1 m/s.

Abbreviations: 95% CI, 95% confidence interval; BMI, body mass index; GDS, Geriatric Depression Scale.

* *p* < 0.05.

** *p* < 0.01.

*** *p* < 0.001.

Higher gait speed (*β* = −0.273, 95% CI = −0.516 to −0.029, *p* < 0.05), handgrip strength (*β* = −0.075, 95% CI = −0.141 to −0.010, *p* < 0.05) and peak leg muscle power (*β* = −0.007, 95% CI = −0.012 to −0.001, *p* < 0.05) significantly attenuated the negative association between age and executive function (Table 3; standardized, per‐SD coefficients are reported in Table S1). Three‐way interaction models revealed a sex‐specific moderating effect of gait speed (*β* = −0.567, 95% CI = −1.060 to −0.073, *p* < 0.05), indicating that the moderating effect of gait speed was specific to men (Table S2). JN analyses indicated that the negative association between age and executive function was statistically detectable below gait speed 1.83 m/s, handgrip strength 48.8 kg and peak leg muscle power 438 W, becoming nonsignificant above these values. Only a small proportion of the sample fell within these upper regions of significance (2.0%, 1.0% and 4.8%, respectively; Figure 1).

**TABLE 3:** Moderation analysis by physical performance model. Interaction‐term coefficients between age and physical performance markers on executive function (TMT‐B minus TMT‐A and Stroop interference) are presented for the whole sample and stratified by sex. Coefficients and 95% CIs are adjusted for socioeconomic status, depressive symptoms, BMI, anxiety, diabetes, hypertension, dyslipidaemia and smoking (plus sex for the whole sample).

Outcome	Physical performance	Whole *β* (95% CI)	*p*	Female *β* (95% CI)	*p*	Male *β* (95% CI)	*p*
TMT B minus A	Gait speed	−0.273 (−0.516 to −0.029)	< 0.05*	0.058 (−0.344 to 0.459)	0.78	−0.514 (−0.821 to −0.207)	< 0.01**
	Handgrip	−0.075 (−0.141 to −0.010)	< 0.05*	−0.005 (−0.202 to 0.193)	0.96	−0.127 (−0.254 to −0.001)	< 0.05*
	Leg muscle power	−0.007 (−0.012 to −0.001)	< 0.05*	−0.005 (−0.021 to 0.011)	0.52	−0.004 (−0.012 to 0.003)	0.28
Stroop Interference	Gait speed	−0.067 (−0.128 to −0.006)	< 0.05*	−0.093 (−0.185 to −0.001)	< 0.05*	−0.027 (−0.112 to 0.058)	0.53
	Handgrip	−0.019 (−0.036 to −0.001)	< 0.05*	−0.016 (−0.065 to 0.033)	0.51	−0.015 (−0.050 to 0.019)	0.39
	Leg muscle power	−0.002 (−0.003 to −0.000)	< 0.05*	−0.002 (−0.006 to 0.002)	0.43	−0.001 (−0.004 to 0.001)	0.2

*Note:* Results correspond to the whole available sample and are also shown as stratified by sex.

Abbreviations: 95% CI, 95% confidence intervals; TMT, Trail Making Test.

* *p* < 0.05.

** *p* < 0.01.

*** *p* < 0.001.

**FIGURE 1:**
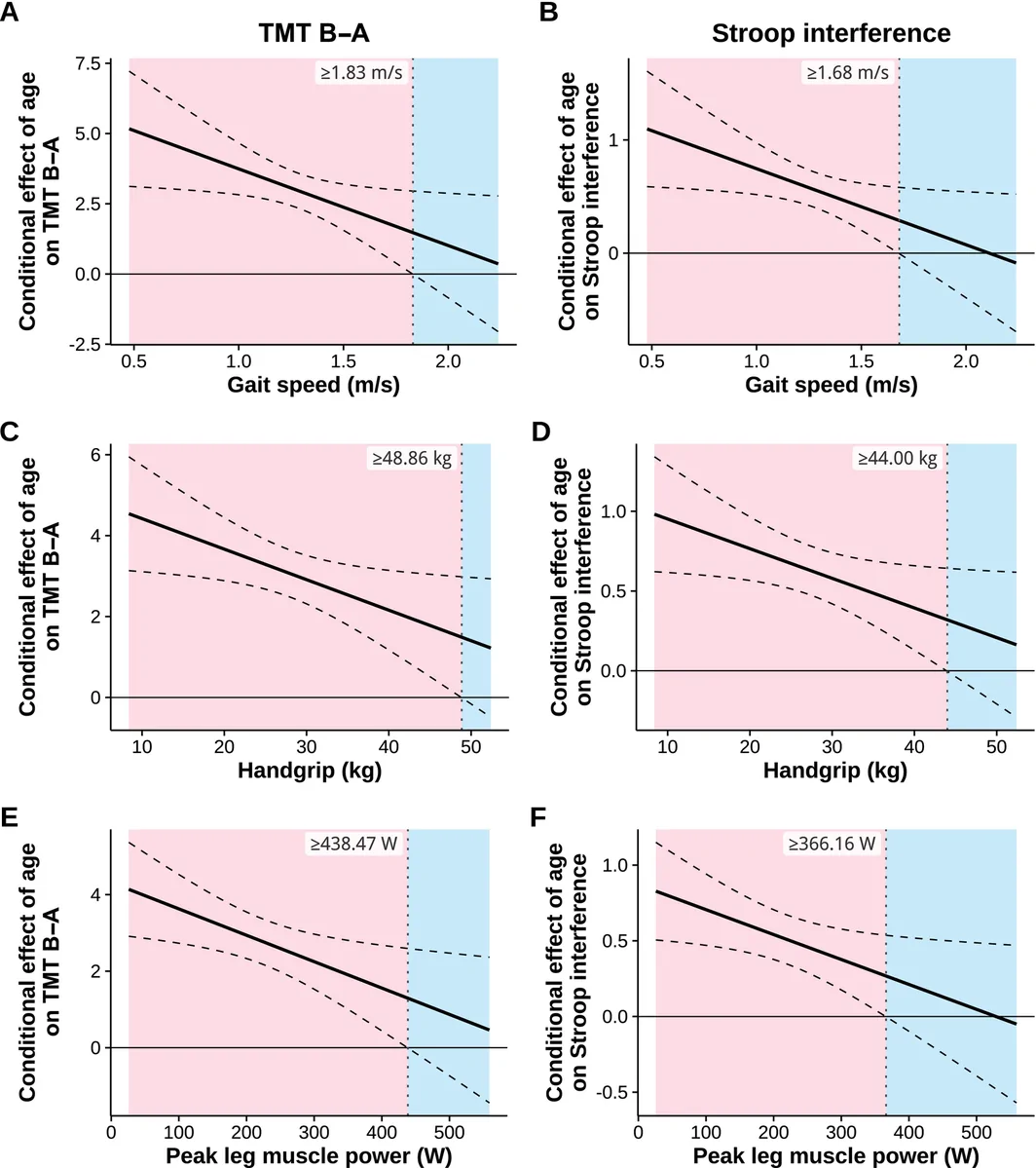
Johnson–Neyman plots delineating gait speed, handgrip strength and peak leg muscle power values where increasing age significantly associates to worse executive function (red area) and values where this association becomes nonsignificant (blue area). Results are presented by cognitive test (TMT B minus A, left column; Stroop Interference, right column). The solid line represents the point estimate of the effect and dotted lines indicate 95% confidence intervals.

#### 3.3.2 Stroop Interference Score

The models testing the moderating role of physical performance in the association between age and Stroop interference scores yielded results consistent with the TMT analyses. Higher gait speed (*β* = −0.067, 95% CI = −0.128 to −0.006, *p* < 0.05), handgrip strength (*β* = −0.019, 95% CI = −0.036 to −0.001, *p* < 0.05) and peak leg muscle power (*β* = −0.002, 95% CI = −0.003 to −0.000, *p* < 0.05) significantly moderated the association between age and executive function. No significant three‐way interactions were observed (all *p* > 0.05; Table S2), indicating no confirmed sex‐specific effect at the interaction level. JN analyses identified regions‐of‐significance below gait speed 1.68 m/s, handgrip strength 43.9 kg, and peak leg muscle power 366 W, with the detrimental effect of age on executive performance becoming nonsignificant above these values. These values corresponded to 5.1%, 6.3% and 15.9% of participants, respectively (Figure 1).

All six primary age × performance interactions remained significant after Benjamini–Hochberg correction for the false discovery rate (all FDR‐adjusted *p* = 0.033).

### 3.4 Subgroup Analyses

#### 3.4.1 Females

Among females, no significant association was found between age and TMT B minus A performance, except in the model including peak leg muscle power, where increasing age was associated with worse outcomes (Table 2). Depressive symptoms and BMI were not independently associated with executive function (all *p* > 0.05). In contrast, higher SES consistently conferred a strong protective effect, with each increment in SES corresponding to a 14.4‐ to 15.6‐s improvement in TMT B minus A performance.

For Stroop interference, age had a significant negative effect, with a borderline deleterious association with depressive symptoms, and SES exerted a consistent protective influence (Tables 2 and 3). In the sex‐stratified models, gait speed significantly moderated the age–Stroop association in females (*β* = −0.093, *p* < 0.05), but not handgrip or leg power (both *p* > 0.05). However, the three‐way age × sex × gait interaction was not significant (*β* = 0.063, *p* = 0.32; Table S2), so this stratified effect should not be interpreted as a true sex‐specific difference.

#### 3.4.2 Males

In males, older age and greater depressive symptoms were significantly associated with poorer executive function. Higher SES conferred strong benefits, with a 10.9‐ to 12.3‐s improvement in TMT B minus A times for each SES increment (Table 2). Multiple regression models revealed a significant moderating effect of gait speed (*β* = −0.514, 95% CI = −0.821 to −0.207, *p* < 0.01) and a weaker but significant effect of handgrip strength (*β* = −0.127, 95% CI = −0.254 to −0.001, *p* < 0.05). Peak leg muscle power did not moderate the association between age and executive functioning (*β* = −0.004, 95% CI = −0.012 to 0.003, *p* = 0.28) (Table 3). JN analyses indicated that the adverse effect of age on executive performance was statistically detectable below a gait speed of 1.59 m/s, becoming nonsignificant above this value, which was reached by 14.0% of males (Figure 2).

**FIGURE 2:**
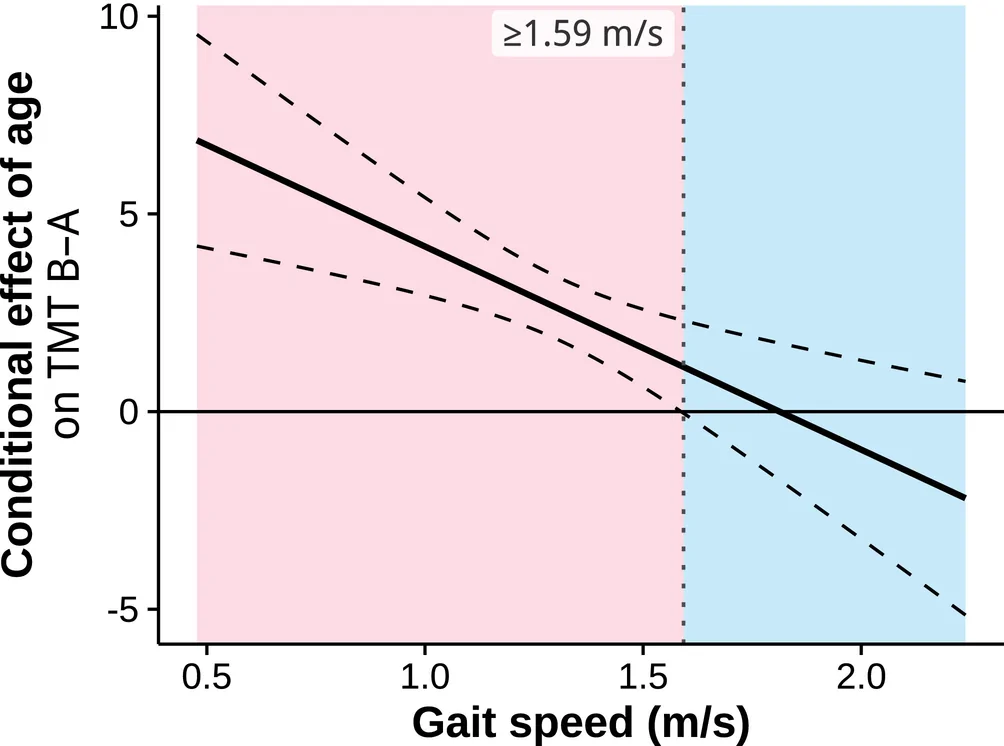
Male subgroup. Johnson–Neyman plot delineating gait speed values where increasing age significantly associates with worse executive function (red area) and values where this association becomes nonsignificant (blue area). The solid line represents the point estimate of the effect, and dotted lines indicate 95% confidence intervals.

Regarding Stroop interference, age and depressive symptoms were significantly associated with worse outcomes in models including leg muscle power. SES conferred robust protection, but no moderating effects of physical performance were identified (Tables 2 and 3).

#### 3.4.3 Role of WMH Burden

Including WMH burden (Fazekas score) as a covariate left the effect‐size estimates for the predictors of both TMT B minus A and Stroop interference essentially unchanged in magnitude and direction. The age × physical‐performance interaction coefficients were likewise similar in size. However, in the WMH‐adjusted subsample, several whole‐sample interaction terms were attenuated to borderline or nonsignificant levels, most plausibly reflecting the reduced sample size with available MRI (*n* = 774 of 920) rather than confounding by WMH. Notably, the moderating effect of gait speed on TMT B minus A in men remained significant (*β* = −0.521, *p* < 0.01) (Tables S3 and S4).

#### 3.4.4 Sensitivity Analyses

Excluding participants with MCI left most marker–outcome associations unchanged, indicating that the broader moderation pattern was not driven by incipient cognitive decline. Two exceptions emerged: the gait effect on TMT B minus A (*β* = −0.191, 95% CI = −0.420 to 0.038, *p* = 0.10) and the handgrip effect on Stroop interference (*β* = −0.016, *p* = 0.07) were attenuated to nonsignificance (Table S5), suggesting that these associations were partly sensitive to the inclusion of participants with early cognitive impairment.

Further adjustment for subjective sleep quality did not materially alter the moderation estimates. For TMT B minus A, all three markers remained significant (gait speed *β* = −0.318, *p* < 0.05; handgrip *β* = −0.078, *p* < 0.05; leg muscle power *β* = −0.007, *p* < 0.05), whereas for Stroop interference estimates were of similar magnitude but attenuated to borderline significance (gait *β* = −0.063, *p* = 0.05; handgrip *β* = −0.016, *p* = 0.08; leg power *β* = −0.001, *p* = 0.05), consistent with the reduced sample size.

Similarly, height‐adjusted gait models were materially unchanged (TMT B minus A, whole sample: *β* = −0.286, *p* = 0.02; men: *β* = −0.541, *p* < 0.001; Stroop interference, whole sample: *β* = −0.069, *p* = 0.03), confirming that the moderating effect of gait speed was not attributable to body size. The female‐specific Stroop effect of gait persisted after height adjustment (*β* = −0.094, *p* = 0.04).

Re‐expressing grip strength and leg power in relative units (kg/BMI and W/kg) attenuated the moderation to nonsignificance in the whole sample, and sex‐stratified relative‐marker models showed no moderation in either females or males (Table S6).

## 4 Discussion

In this large population‐based cohort of community‐dwelling older adults at increased risk for dementia, higher gait speed, handgrip strength and leg muscle power were associated with a weaker relationship between increasing age and poorer executive function, independent of SES, depressive and anxiety symptoms, BMI and cardiometabolic alterations. This moderation was not uniform across sexes resulting most evident for gait speed in males, a pattern we examine below. Using JN analyses, we delineated regions of significance indicating the range of performance values above which the age–executive function association became statistically undetectable. These values fell at the upper extreme of the performance distribution and were reached by only a small proportion of participants, pointing to a high‐reserve neuromuscular phenotype and its relationship with better executive function.

Analyses using a literacy‐independent executive function measure (Stroop interference score) supported the robustness of our findings, extending them to a larger subsample. Participants included in each analysis did not differ substantially from those excluded by missing outcome or physical‐performance data (Tables S7 and S8). Notably, the JN regions‐of‐significance extended to lower performance values in the Stroop‐based models, indicating that a greater proportion of participants fell within the range where age was not statistically associated with executive function, particularly for inhibitory control. Importantly, JN points should be interpreted as sample‐specific regions‐of‐significance rather than actionable clinical cutoffs, as their location depends on the moderator distribution, residual variance and sample size.

Sex modulated the findings for gait speed. For TMT B minus A, the moderating effect of gait was confined to males and supported by a significant three‐way interaction. For the Stroop task, by contrast, gait moderated the association within the female stratum, but the corresponding three‐way interaction was not significant. This divergence should not be over‐interpreted as a true sex‐specific effect and more likely reflects endpoint‐specific dynamics that warrant further investigation into the biological, behavioural and social factors involved.

Sensitivity analyses limited to cognitively unimpaired individuals (excluding participants with MCI) left most marker–outcome associations unchanged, indicating that the broader moderation pattern was not driven by incipient cognitive decline. The exceptions were the gait effect on TMT B minus A and the handgrip effect on Stroop interference, which were attenuated to nonsignificance. This suggests that gait speed may be a particularly sensitive marker of executive decline in individuals with established MCI [47].

Our findings expand the increasing body of research linking physical performance to executive function during aging. Meta‐analyses of cross‐sectional studies reveal small but consistent associations between faster gait speed and better executive function, although with notable variability [48, 49]. Longitudinal data also show that lower gait speed [50, 51] and weaker handgrip strength [52] predict faster cognitive decline, even after adjusting for sociodemographic factors, health behaviours and clinical variables. Similarly, lower baseline muscle power has been associated with a 10‐year decline in overall cognition in twin studies, indicating a potential motor‐cognitive connection independent of genetics and early‐life environment [53]. Despite this increasing evidence, no previous study has investigated the moderating effects, delineated the corresponding regions‐of‐significance or examined sex‐specific differences in the relationship between aging, physical performance and executive function in older adults at risk for dementia.

A recent large‐scale longitudinal study involving nearly 86 000 adults over 9.3 years found that higher handgrip strength was linked to a lower incidence of AD, with optimal protective thresholds appearing at very high strength levels (e.g., 49 kg) [54]. This value is close to the regions‐of‐significance identified in our handgrip models but was markedly higher than the cutoffs for low muscle strength proposed by international sarcopenia groups (e.g., < 27 kg for men and < 16 kg for women, as per EWGSOP2). While those lower cutoffs reliably identify vulnerability to functional decline and early cognitive impairment, our results suggest that substantially higher physical performance is associated with a nondetectable age‐executive function relationship in individuals with an elevated long‐term risk, as indicated by the CAIDE dementia risk score.

Evidence from combined US and European cohorts shows that declines in handgrip strength and gait speed accelerate 6–8 years before dementia onset [55], mirroring reports of frailty acceleration 4–9 years prior to diagnosis [56]. These findings suggest that sarcopenia and frailty could serve as midterm indicators of vulnerability, while exceptionally high physical performance might provide resilience earlier in the process of cognitive deterioration. Confirming this framework and characterizing the performance ranges associated with attenuated age‐related executive vulnerability could help establish early preventive goals.

Previous trials within the World Wide FINGERS (WW‐FINGERS) initiative have shown that intensive lifestyle interventions improve overall cognition in older adults at risk for dementia, with lasting gains in processing speed and executive function [57, 58]. However, the mechanisms behind this effect remain unclear. The FINGER multidomain intervention reduced the risk of cardiac events, stroke and transient ischemic attacks (TIAs), indicating a possible mediating role of vascular pathways in cognitive benefits [59]. Nonetheless, neuroimaging substudies of the original FINGER trial found no significant differences in the progression of white matter lesions, suggesting that cerebrovascular changes alone may not explain the observed improvements [60]. In contrast, the SPRINT‐MIND trial demonstrated that intensive blood pressure control significantly slowed WMH progression and reduced the risk of mild cognitive impairment, supporting the vascular contribution hypothesis [61, 62].

In our cross‐sectional analysis of the CITA GO‐ON subsample with available baseline MRI data, WMH burden was not independently associated with executive function after adjusting for key covariates, including cognitive reserve and depressive symptoms. Although our results differ from previous studies linking WMH burden to executive dysfunction, they should not be seen as disproving these well‐established relationships. Instead, several methodological factors likely explain this difference. First, the WMH burden was assessed using the Fazekas scale, a semiquantitative visual rating method that is less sensitive than volumetric measurements or diffusion tensor imaging (DTI) for detecting subtle cerebrovascular pathology [63]. Moreover, periventricular and deep white‐matter components were not recorded separately, precluding region‐specific analyses, despite evidence that executive function may track periventricular burden more closely. Second, the CITA GO‐ON sample may not fully represent populations with greater cognitive impairment, where WMH have stronger links to cognition. Finally, the MRI subsample comprised 774 of 920 participants. Those without MRI‐derived WMH data did not apparently differ in age, sex or cognitive status, although they had marginally higher educational attainment (Table S9). Given potential selection into MRI, WMH‐adjusted models should be interpreted as conservative estimates. Overall, these limitations underscore the need for more accurate neuroimaging techniques to better understand the role of WMH in age‐related cognitive decline. Future studies should employ volumetric or DTI‐based methods, differentiate between periventricular and deep WMH and include longitudinal designs to clarify how vascular burden, lesion location, physical performance and cognitive resilience interact across the dementia spectrum. Within this framework, our results suggest that higher neuromuscular performance may be associated with reduced detectability of age‐related executive vulnerability, offering a potential functional‐reserve pathway that could complement vascular‐risk optimisation in multidomain prevention programmes. The pragmatic performance targets mapping onto cognitive resilience remain to be defined.

Beyond vascular factors, neurodegenerative burden, including Alzheimer's disease and alpha‐synucleinopathies, greatly impacts cognitive trajectories and remains a less explored aspect in nonpharmacological, multidomain dementia prevention trials. Ongoing efforts in CITA GO‐ON and related studies, which include biological sampling and neuroimaging, will be key to understanding the interactions among physical performance, AD biomarkers, cerebrovascular burden and cognition.

The cognitive reserve hypothesis proposes that lifelong experiences like education, occupation and socioeconomic status strengthen the brain's ability to maintain cognitive performance despite ongoing neuropathology [42]. In our study, socioeconomic status (SES) was the second most significant independent factor influencing executive functioning, after chronological age. Each higher SES level was associated with an approximate 11‐ to 15‐s reduction in TMT B minus A completion times, consistent across sexes. Given the importance of processing speed and executive function improvements to the success of modern nondrug dementia prevention trials, and the notable link between SES and the age–executive function relationship highlighted here, future prevention studies should include SES‐stratified analyses. These approaches could help optimize resource allocation and ensure that individuals with lower cognitive reserve receive appropriately targeted support, thereby enhancing the effectiveness of population‐wide interventions.

To our knowledge, this is the first study to explore the moderating role of three distinct physical performance domains on the relationship between age and executive function, utilizing a large, well‐characterized, population‐based sample of older adults at risk of dementia and thoroughly controlling for cognitive reserve and mood‐related covariates. Another strength of this study is the disaggregated analysis of sex‐specific effects and the specific impact of WMH, which enhances our understanding and guides future research. Finally, delineating the regions‐of‐significance for this moderation in a vulnerable sample is a novel contribution. However, despite the consistency of our findings, these regions were located at the extreme of the performance distribution and may be practically difficult to reach through exercise or lifestyle interventions, particularly when initiated later in life.

The principal contribution of this study is to move beyond whether physical performance correlates with executive function and instead to test whether physical performance changes the strength of the age–executive function association. By applying moderation modelling with JN probing in a large at‐risk cohort and triangulating results across two executive measures, we provide a reproducible analytic template that can be applied across WW‐FINGERS cohorts and other sarcopenia and brain‐aging datasets.

This study had several limitations. First, the cross‐sectional design is inherently prone to bias and cannot establish causality, making these findings hypothesis‐generating. Second, although vascular comorbidities and smoking were adjusted for, residual confounding remains possible, as medication exposure and habitual physical activity, which may jointly influence physical performance and executive outcomes, were not comprehensively modelled. Third, the results may not be applicable to other non‐Western, lower SES or cognitively impaired populations due to cultural, social and clinical differences. Fourth, despite no clear differences across participants with and without MRI data, the limited number of MRI scans and the absence of volumetric WMH quantification only allow for preliminary insights into the relationship between WMH, physical performance and executive function. Lastly, biochemical or neuroimaging biomarkers were not readily available to assess neuropathological burden, determine the aetiology of prodromal stages of neurodegenerative diseases or characterize critical, nonmodifiable risk factors like the APOE genotype, which affected our conclusions. Future research should examine whether physical performance influences the association between age and executive function in ways that depend on WMH volume, neuropathology burden or APOE status.

In conclusion, physical performance moderates the relationship between age and executive function in community‐dwelling older adults at increased risk of dementia. Higher physical performance, most consistently gait speed and primarily in males, was associated with a weaker link between age and executive function. Our findings also highlight the influence of cognitive reserve–related factors, particularly socioeconomic status, in supporting executive function in later life. Long‐term studies using sensitive neuroimaging techniques, as well as biologically characterized and diverse populations are necessary to verify these results, establish whether any performance range translates into practical targets, investigate underlying mechanisms and evaluate the effectiveness of performance‐based preventive strategies.

## Supporting information


**Figure S1:** Flow chart of participants and analytic samples. Analytic *N* values correspond to the complete‐case sample available for each age × physical‐performance interaction model after listwise deletion of missing outcome, predictor, moderator and covariate data (socioeconomic status, depressive symptoms, body mass index, sex, anxiety, diabetes, hypertension, dyslipidaemia and smoking).

**Table S1:** Standardized interaction coefficients (per SD). *z*‐scores were computed on the full sample so estimates are comparable across sex and markers.

**Table S2:** Three‐way interaction (age × sex × physical performance) on executive function. Adjusted for socioeconomic status, GDS, BMI, anxiety, diabetes, hypertension, dyslipidaemia and smoking.

**Table S3:** Multiple regression additionally adjusted for white‐matter hyperintensities (Fazekas). Centred, raw‐unit betas; gait speed per 0.1 m/s.

**Table S4:** Moderation (age × performance) additionally adjusted for white‐matter hyperintensities (Fazekas).

**Table S5:** Sensitivity analysis in cognitively unimpaired participants (mild cognitive impairment excluded). Moderation (age × performance) on executive function.

**Table S6:** Relative‐marker sensitivity analyses for the age × physical‐performance moderation. Handgrip and leg power were re‐expressed in relative units (kg/BMI and W/kg). To avoid double adjustment for body size, body mass index was not included as a covariate in these models. Models adjusted for socioeconomic status, GDS, sex, anxiety, diabetes, hypertension, dyslipidaemia and smoking. Raw (centred) and per‐SD standardised coefficients are shown. Sex‐stratified raw coefficients are also shown.

**Table S7:** Comparison of participants included versus excluded by each executive outcome (TMT‐B minus TMT‐A and Stroop interference).

**Table S8:** Comparison of participants with versus without physical performance data.

**Table S9:** Comparison of participants with versus without MRI‐derived WMH data.
