# Supplementary material for: Physical Performance Moderates the Association Between Age and Executive Function in Older Adults at Elevated Risk of Dementia: Cross‐Sectional Analysis From the CITA GO‐ON Trial
Source: J Cachexia Sarcopenia Muscle. 2026 Oct 5;17(5):e70396. doi: 10.1002/jcsm.70396 (PMC13638253; doi:10.1002/jcsm.70396)
Supplement: Supplementary file 1 [file JCSM-17-e70396-s001.docx]

Supplementary Table 1. Standardized interaction coefficients (per SD). z-scores were computed on the full sample so estimates are comparable across sex and markers.

| **Outcome** | **Physical performance** | **Whole Std. β (per SD, 95% CI)** | **p** | **Women Std. β (per SD, 95% CI)** | **p** | **Men Std. β (per SD, 95% CI)** | **p** |
| --- | --- | --- | --- | --- | --- | --- | --- |
| TMT B minus A | Gait Speed | -3.863 (-7.309–-0.416) | **<0.05*** | 0.816 (-4.867–6.499) | 0.78 | -7.280 (-11.626–-2.933) | **<0.01**** |
|  | Handgrip | -4.119 (-7.705–-0.532) | **<0.05*** | -0.263 (-11.056–10.530) | 0.96 | -6.951 (-13.861–-0.040) | **<0.05*** |
|  | Leg Muscle Power | -4.509 (-8.046–-0.972) | **<0.05*** | -3.420 (-13.868–7.029) | 0.52 | -2.804 (-7.861–2.253) | 0.28 |
| Stroop Interference | Gait Speed | -0.951 (-1.813–-0.089) | **<0.05*** | -1.322 (-2.626–-0.018) | **<0.05*** | -0.386 (-1.589–0.817) | 0.53 |
|  | Handgrip | -1.016 (-1.951–-0.081) | **<0.05*** | -0.893 (-3.569–1.784) | 0.51 | -0.826 (-2.709–1.058) | 0.39 |
|  | Leg Muscle Power | -1.077 (-2.026–-0.129) | **<0.05*** | -1.039 (-3.628–1.550) | 0.43 | -0.957 (-2.429–0.515) | 0.2 |

95% CI, 95% confidence interval. *p<0.05; **p<0.01; ***p<0.001
