# Supplementary material for: Physical Performance Moderates the Association Between Age and Executive Function in Older Adults at Elevated Risk of Dementia: Cross‐Sectional Analysis From the CITA GO‐ON Trial
Source: J Cachexia Sarcopenia Muscle. 2026 Oct 5;17(5):e70396. doi: 10.1002/jcsm.70396 (PMC13638253; doi:10.1002/jcsm.70396)
Supplement: Supplementary file 2 [file JCSM-17-e70396-s002.docx]

Supplementary Table 5. Sensitivity analysis in cognitively unimpaired participants (mild cognitive impairment excluded). Moderation (age × performance) on executive function.

| **Outcome** | **Physical performance** | **β (95% CI)** | **p** |
| --- | --- | --- | --- |
| TMT B minus A | Gait Speed | -0.191 (-0.420–0.038) | 0.1 |
|  | Handgrip | -0.073 (-0.132–-0.015) | <0.05* |
|  | Leg Muscle Power | -0.005 (-0.010–0.000) | 0.05 |
| Stroop Interference | Gait Speed | -0.070 (-0.131–-0.008) | <0.05* |
|  | Handgrip | -0.016 (-0.033–0.001) | 0.07 |
|  | Leg Muscle Power | -0.002 (-0.003–-0.000) | <0.05* |

*95% CI, 95% confidence interval. *p<0.05; **p<0.01; ***p<0.001*
