# Supplementary material for: Physical Performance Moderates the Association Between Age and Executive Function in Older Adults at Elevated Risk of Dementia: Cross‐Sectional Analysis From the CITA GO‐ON Trial
Source: J Cachexia Sarcopenia Muscle. 2026 Oct 5;17(5):e70396. doi: 10.1002/jcsm.70396 (PMC13638253; doi:10.1002/jcsm.70396)
Supplement: Supplementary file 3 [file JCSM-17-e70396-s003.docx]

Supplementary Table 3. Multiple regression additionally adjusted for white-matter hyperintensities (Fazekas). Centered, raw-unit betas; gait speed per 0.1 m/s.

| **Outcome** | **Predictor** | **Performance model** | **Whole β (95% CI)** | **p** | **Female β (95% CI)** | **p** | **Male β (95% CI)** | **p** |
| --- | --- | --- | --- | --- | --- | --- | --- | --- |
| TMT B minus A | Age (years) | Gait Speed | 2.75 (2.06–3.44) | <0.001*** | 3.10 (2.03–4.17) | <0.001*** | 2.53 (1.57–3.49) | <0.001*** |
|  |  | Handgrip | 2.80 (2.11–3.49) | <0.001*** | 2.85 (0.85–4.84) | <0.01** | 3.24 (1.94–4.55) | <0.001*** |
|  |  | Leg Muscle Power | 2.67 (1.98–3.36) | <0.001*** | 2.66 (0.90–4.42) | <0.01** | 2.38 (1.31–3.44) | <0.001*** |
|  | Sex (ref. female) | Gait Speed | 4.30 (-3.58–12.18) | 0.28 | - | - | - | - |
|  |  | Handgrip | 10.87 (-1.90–23.64) | 0.1 | - | - | - | - |
|  |  | Leg Muscle Power | 7.60 (-3.35–18.55) | 0.17 | - | - | - | - |
|  | Socioeconomic Status Index | Gait Speed | -13.01 (-16.63–-9.40) | <0.001*** | -12.53 (-17.68–-7.37) | <0.001*** | -11.79 (-17.15–-6.42) | <0.001*** |
|  |  | Handgrip | -13.27 (-16.91–-9.62) | <0.001*** | -12.81 (-17.97–-7.65) | <0.001*** | -12.27 (-17.70–-6.84) | <0.001*** |
|  |  | Leg Muscle Power | -13.67 (-17.25–-10.09) | <0.001*** | -12.53 (-17.91–-7.15) | <0.001*** | -13.31 (-18.33–-8.30) | <0.001*** |
|  | Depression (GDS) | Gait Speed | 1.82 (-0.01–3.66) | 0.05 | 0.93 (-1.69–3.54) | 0.48 | 3.05 (0.37–5.74) | <0.05* |
|  |  | Handgrip | 2.03 (0.20–3.85) | <0.05* | 1.11 (-1.50–3.72) | 0.4 | 3.20 (0.49–5.92) | <0.05* |
|  |  | Leg Muscle Power | 2.05 (0.27–3.84) | <0.05* | 1.02 (-1.69–3.73) | 0.46 | 3.70 (1.22–6.19) | <0.01** |
|  | Anxiety | Gait Speed | 0.68 (-0.99–2.35) | 0.42 | 1.12 (-1.25–3.49) | 0.35 | -0.12 (-2.58–2.35) | 0.93 |
|  |  | Handgrip | 0.80 (-0.87–2.47) | 0.35 | 1.23 (-1.15–3.62) | 0.31 | 0.08 (-2.41–2.57) | 0.95 |
|  |  | Leg Muscle Power | 0.79 (-0.85–2.42) | 0.34 | 1.35 (-1.12–3.82) | 0.28 | -0.27 (-2.56–2.02) | 0.82 |
|  | BMI | Gait Speed | -0.72 (-1.57–0.13) | 0.1 | -0.90 (-2.05–0.25) | 0.13 | -0.59 (-1.95–0.77) | 0.39 |
|  |  | Handgrip | -0.46 (-1.30–0.38) | 0.28 | -0.53 (-1.63–0.56) | 0.34 | -0.52 (-1.93–0.88) | 0.47 |
|  |  | Leg Muscle Power | -0.30 (-1.15–0.54) | 0.48 | -0.22 (-1.40–0.97) | 0.72 | -0.49 (-1.80–0.83) | 0.47 |
|  | Diabetes | Gait Speed | 2.82 (-10.15–15.79) | 0.67 | 0.34 (-19.87–20.54) | 0.97 | 4.67 (-12.37–21.71) | 0.59 |
|  |  | Handgrip | 2.40 (-10.59–15.40) | 0.72 | 0.82 (-19.49–21.13) | 0.94 | 2.26 (-14.90–19.42) | 0.8 |
|  |  | Leg Muscle Power | 1.48 (-11.60–14.56) | 0.82 | -1.17 (-22.11–19.77) | 0.91 | 2.68 (-14.04–19.40) | 0.75 |
|  | Hypertension | Gait Speed | -3.88 (-11.43–3.67) | 0.31 | -3.76 (-15.15–7.63) | 0.52 | -5.22 (-15.47–5.04) | 0.32 |
|  |  | Handgrip | -4.15 (-11.76–3.46) | 0.28 | -3.72 (-15.14–7.70) | 0.52 | -4.97 (-15.41–5.48) | 0.35 |
|  |  | Leg Muscle Power | -3.01 (-10.47–4.45) | 0.43 | -3.76 (-15.51–8.00) | 0.53 | -1.89 (-11.64–7.86) | 0.7 |
|  | Dyslipidaemia | Gait Speed | 0.48 (-6.86–7.81) | 0.9 | -0.72 (-11.95–10.52) | 0.9 | 1.21 (-8.70–11.12) | 0.81 |
|  |  | Handgrip | 0.53 (-6.85–7.92) | 0.89 | 0.30 (-11.07–11.68) | 0.96 | 1.30 (-8.76–11.37) | 0.8 |
|  |  | Leg Muscle Power | 1.12 (-6.13–8.37) | 0.76 | -1.61 (-13.29–10.06) | 0.79 | 3.88 (-5.52–13.29) | 0.42 |
|  | Smoking: Current (ref. Never) | Gait Speed | -6.39 (-21.03–8.26) | 0.39 | -13.53 (-37.85–10.79) | 0.27 | -0.56 (-19.17–18.06) | 0.95 |
|  |  | Handgrip | -5.81 (-20.64–9.02) | 0.44 | -13.68 (-38.12–10.76) | 0.27 | 0.23 (-18.81–19.26) | 0.98 |
|  |  | Leg Muscle Power | -4.33 (-18.75–10.09) | 0.56 | -13.07 (-38.32–12.18) | 0.31 | 2.44 (-15.04–19.92) | 0.78 |
|  | Smoking: Former (ref. Never) | Gait Speed | -5.89 (-13.51–1.74) | 0.13 | -10.59 (-22.52–1.35) | 0.08 | 0.00 (-10.30–10.30) | 1 |
|  |  | Handgrip | -5.28 (-12.97–2.41) | 0.18 | -10.89 (-22.90–1.12) | 0.08 | 0.19 (-10.24–10.61) | 0.97 |
|  |  | Leg Muscle Power | -4.37 (-11.87–3.13) | 0.25 | -10.23 (-22.61–2.15) | 0.11 | 2.08 (-7.60–11.77) | 0.67 |
|  | WMH (Fazekas) | Gait Speed | 2.18 (-2.66–7.02) | 0.38 | -1.31 (-8.85–6.23) | 0.73 | 5.29 (-1.10–11.67) | 0.1 |
|  |  | Handgrip | 2.13 (-2.72–6.99) | 0.39 | -1.64 (-9.18–5.89) | 0.67 | 5.37 (-1.09–11.83) | 0.1 |
|  |  | Leg Muscle Power | 0.33 (-4.44–5.10) | 0.89 | -1.49 (-9.24–6.26) | 0.71 | 1.80 (-4.24–7.85) | 0.56 |
| Stroop Interference | Age (years) | Gait Speed | 0.53 (0.35–0.71) | <0.001*** | 0.52 (0.24–0.80) | <0.001*** | 0.49 (0.24–0.74) | <0.001*** |
|  |  | Handgrip | 0.59 (0.41–0.76) | <0.001*** | 0.54 (0.04–1.05) | <0.05* | 0.56 (0.23–0.89) | <0.001*** |
|  |  | Leg Muscle Power | 0.50 (0.32–0.69) | <0.001*** | 0.44 (-0.02–0.89) | 0.06 | 0.46 (0.17–0.75) | <0.01** |
|  | Sex (ref. female) | Gait Speed | 0.60 (-1.45–2.65) | 0.56 | - | - | - | - |
|  |  | Handgrip | 2.09 (-1.27–5.45) | 0.22 | - | - | - | - |
|  |  | Leg Muscle Power | 3.53 (0.57–6.48) | <0.05* | - | - | - | - |
|  | Socioeconomic Status Index | Gait Speed | -3.38 (-4.30–-2.46) | <0.001*** | -3.23 (-4.57–-1.88) | <0.001*** | -3.23 (-4.56–-1.91) | <0.001*** |
|  |  | Handgrip | -3.47 (-4.39–-2.54) | <0.001*** | -3.28 (-4.63–-1.93) | <0.001*** | -3.35 (-4.68–-2.02) | <0.001*** |
|  |  | Leg Muscle Power | -3.34 (-4.28–-2.40) | <0.001*** | -3.16 (-4.57–-1.76) | <0.001*** | -3.18 (-4.51–-1.85) | <0.001*** |
|  | Depression (GDS) | Gait Speed | 0.40 (-0.08–0.87) | 0.1 | 0.39 (-0.28–1.07) | 0.25 | 0.50 (-0.19–1.19) | 0.15 |
|  |  | Handgrip | 0.45 (-0.03–0.92) | 0.06 | 0.43 (-0.24–1.11) | 0.21 | 0.57 (-0.12–1.26) | 0.11 |
|  |  | Leg Muscle Power | 0.48 (0.01–0.96) | <0.05* | 0.43 (-0.26–1.12) | 0.22 | 0.69 (-0.00–1.38) | 0.05 |
|  | Anxiety | Gait Speed | -0.07 (-0.49–0.35) | 0.75 | -0.04 (-0.64–0.55) | 0.88 | -0.20 (-0.83–0.44) | 0.54 |
|  |  | Handgrip | -0.05 (-0.48–0.37) | 0.81 | -0.03 (-0.63–0.57) | 0.91 | -0.18 (-0.82–0.45) | 0.58 |
|  |  | Leg Muscle Power | -0.02 (-0.45–0.41) | 0.93 | -0.00 (-0.62–0.62) | 1 | -0.15 (-0.78–0.48) | 0.63 |
|  | BMI | Gait Speed | -0.09 (-0.31–0.13) | 0.42 | -0.15 (-0.46–0.16) | 0.34 | -0.08 (-0.43–0.28) | 0.66 |
|  |  | Handgrip | -0.03 (-0.25–0.19) | 0.79 | -0.05 (-0.34–0.25) | 0.76 | -0.05 (-0.41–0.31) | 0.79 |
|  |  | Leg Muscle Power | 0.01 (-0.22–0.24) | 0.91 | 0.02 (-0.29–0.33) | 0.89 | -0.03 (-0.39–0.34) | 0.89 |
|  | Diabetes | Gait Speed | 2.43 (-0.82–5.67) | 0.14 | 2.20 (-2.83–7.24) | 0.39 | 2.41 (-1.87–6.70) | 0.27 |
|  |  | Handgrip | 2.33 (-0.92–5.59) | 0.16 | 2.24 (-2.82–7.30) | 0.39 | 2.26 (-2.03–6.56) | 0.3 |
|  |  | Leg Muscle Power | 2.16 (-1.19–5.52) | 0.21 | 2.47 (-2.67–7.61) | 0.34 | 1.74 (-2.73–6.20) | 0.45 |
|  | Hypertension | Gait Speed | -0.08 (-2.04–1.89) | 0.94 | 0.32 (-2.64–3.27) | 0.83 | -0.36 (-3.05–2.34) | 0.8 |
|  |  | Handgrip | -0.15 (-2.14–1.83) | 0.88 | 0.31 (-2.66–3.28) | 0.84 | -0.54 (-3.26–2.19) | 0.7 |
|  |  | Leg Muscle Power | -0.30 (-2.31–1.70) | 0.77 | 0.19 (-2.85–3.23) | 0.9 | -0.70 (-3.42–2.02) | 0.61 |
|  | Dyslipidaemia | Gait Speed | 0.32 (-1.58–2.21) | 0.74 | -0.53 (-3.44–2.38) | 0.72 | 1.03 (-1.55–3.60) | 0.43 |
|  |  | Handgrip | 0.42 (-1.49–2.34) | 0.66 | -0.38 (-3.31–2.56) | 0.8 | 1.13 (-1.47–3.72) | 0.39 |
|  |  | Leg Muscle Power | 0.22 (-1.72–2.16) | 0.82 | -1.13 (-4.13–1.88) | 0.46 | 1.47 (-1.13–4.07) | 0.27 |
|  | Smoking: Current (ref. Never) | Gait Speed | 0.46 (-3.33–4.24) | 0.81 | -2.63 (-9.07–3.81) | 0.42 | 2.56 (-2.19–7.31) | 0.29 |
|  |  | Handgrip | 0.67 (-3.17–4.51) | 0.73 | -2.72 (-9.21–3.77) | 0.41 | 2.82 (-2.00–7.64) | 0.25 |
|  |  | Leg Muscle Power | 0.90 (-2.95–4.75) | 0.65 | -2.21 (-8.85–4.42) | 0.51 | 2.87 (-1.89–7.63) | 0.24 |
|  | Smoking: Former (ref. Never) | Gait Speed | -0.64 (-2.63–1.35) | 0.53 | -1.42 (-4.54–1.70) | 0.37 | 0.33 (-2.36–3.02) | 0.81 |
|  |  | Handgrip | -0.50 (-2.51–1.51) | 0.63 | -1.59 (-4.72–1.55) | 0.32 | 0.60 (-2.10–3.30) | 0.66 |
|  |  | Leg Muscle Power | -0.81 (-2.83–1.22) | 0.43 | -1.88 (-5.09–1.34) | 0.25 | 0.32 (-2.36–3.01) | 0.81 |
|  | WMH (Fazekas) | Gait Speed | 0.82 (-0.44–2.08) | 0.2 | 0.68 (-1.26–2.62) | 0.49 | 0.95 (-0.73–2.63) | 0.27 |
|  |  | Handgrip | 0.70 (-0.56–1.97) | 0.28 | 0.62 (-1.33–2.56) | 0.53 | 0.80 (-0.89–2.49) | 0.35 |
|  |  | Leg Muscle Power | 0.43 (-0.84–1.71) | 0.5 | 0.50 (-1.48–2.48) | 0.62 | 0.40 (-1.29–2.10) | 0.64 |

*WMH, white-matter hyperintensities; 95% CI, 95% confidence interval. *p<0.05; **p<0.01; ***p<0.001*
