# Supplementary material for: Physical Performance Moderates the Association Between Age and Executive Function in Older Adults at Elevated Risk of Dementia: Cross‐Sectional Analysis From the CITA GO‐ON Trial
Source: J Cachexia Sarcopenia Muscle. 2026 Oct 5;17(5):e70396. doi: 10.1002/jcsm.70396 (PMC13638253; doi:10.1002/jcsm.70396)
Supplement: Supplementary file 4 [file JCSM-17-e70396-s004.docx]

Supplementary Table 6. Relative-marker sensitivity analyses for the age × physical-performance moderation. Handgrip and leg power were re-expressed in relative units (kg/BMI and W/kg). To avoid double adjustment for body size, body mass index was not included as a covariate in these models. Models adjusted for socioeconomic status, GDS, sex, anxiety, diabetes, hypertension, dyslipidaemia and smoking. Raw (centered) and per-SD standardised coefficients are shown. Sex-stratified raw coefficients are also shown.

| **Outcome** | **Physical performance (relative)** | **Age × marker β (95% CI), raw** | **p** | **Std. β (per SD, 95% CI)** | **p** | **Female β (95% CI)** | **p** | **Male β (95% CI)** | **p** |  |  |
| --- | --- | --- | --- | --- | --- | --- | --- | --- | --- | --- | --- |
| TMT B minus A | Handgrip (kg/BMI) | -0.833 (-2.486–0.820) | 0.32 | -1.749 (-5.219–1.721) | 0.32 | 0.199 (-3.37–3.77) | 0.91 | -0.264 (-3.39–2.87) | 0.87 |  |  |
|  | Leg power (W/kg) | -0.355 (-0.828–0.117) | 0.14 | -2.538 (-5.912–0.836) | 0.14 | -0.099 (-1.21–1.01) | 0.86 | -0.109 (-0.75–0.53) 0.74 | | |  |
| Stroop Interference | Handgrip (kg/BMI) | -0.358 (-0.791–0.075) | 0.10 | -0.751 (-1.660–0.157) | 0.10 | -0.227 (-1.12,–0.67) | 0.62 | -0.164 (-1.00–0.67) | 0.70 |  |  |
|  | Leg power (W/kg) | -0.096 (-0.224–0.032) | 0.14 | -0.684 (-1.597–0.229) | 0.14 | -0.013 (-0.29–0.27) | 0.93 | -0.102 (-0.29–0.08) | 0.28 |  |  |

*95% CI, 95% confidence interval. *p<0.05; **p<0.01; ***p<0.001*
