# Supplementary material for: Physical Performance Moderates the Association Between Age and Executive Function in Older Adults at Elevated Risk of Dementia: Cross‐Sectional Analysis From the CITA GO‐ON Trial
Source: J Cachexia Sarcopenia Muscle. 2026 Oct 5;17(5):e70396. doi: 10.1002/jcsm.70396 (PMC13638253; doi:10.1002/jcsm.70396)
Supplement: Supplementary file 5 [file JCSM-17-e70396-s005.docx]

Supplementary Table 7. Comparison of participants included versus excluded by each executive outcome (TMT-B minus TMT-A and Stroop interference).

|  | **TMT-B minus TMT-A** | | | **Stroop interference** | | |
| --- | --- | --- | --- | --- | --- | --- |
| **Characteristic** | **Excluded**  N = 79 | **Included**  N = 841 | **p-value** | **Excluded**  N = 13 | **Included**  N = 907 | **p-value** |
| Age, years | 73.8 (6.3) | 69.1 (6.0) | <0.001*** | 73.8 (6.2) | 69.5 (6.2) | <0.05* |
| Sex |  |  | <0.01** |  |  | <0.05* |
| Women | 52 (66%) | 393 (47%) |  | 2 (15%) | 443 (49%) |  |
| Men | 27 (34%) | 448 (53%) |  | 11 (85%) | 464 (51%) |  |
| Education level, years | 8.5 (3.3) | 13.4 (4.3) | <0.001*** | 14.8 (3.9) | 13.0 (4.5) | 0.10 |
| Socioeconomic Status Index | 0.6 (0.9) | 1.9 (1.1) | <0.001*** | 2.4 (1.0) | 1.8 (1.1) | 0.055 |
| Syndromic Diagnosis |  |  | <0.001*** |  |  | <0.001*** |
| Cognitively Healthy | 46 (58%) | 760 (90%) |  | 7 (54%) | 799 (88%) |  |
| Subjective Cognitive Decline | 4 (5.1%) | 47 (5.6%) |  | 0 (0%) | 51 (5.6%) |  |
| Mild Cognitive Impairment | 29 (37%) | 34 (4.0%) |  | 6 (46%) | 57 (6.3%) |  |
| Body Mass Index | 26.3 (4.6) | 27.5 (4.7) | <0.05* | 27.0 (6.5) | 27.4 (4.7) | 0.3 |
| Fazekas Scale, 0-3 |  |  | 0.5 |  |  | 0.6 |
| 0 | 11 (17%) | 155 (22%) |  | 2 (25%) | 164 (21%) |  |
| 1 | 30 (47%) | 351 (49%) |  | 3 (38%) | 378 (49%) |  |
| 2 | 21 (33%) | 176 (25%) |  | 2 (25%) | 195 (25%) |  |
| 3 | 2 (3.1%) | 28 (3.9%) |  | 1 (13%) | 29 (3.8%) |  |
| GDS score | 2.1 (2.1) | 1.9 (2.2) | 0.3 | 1.7 (2.1) | 1.9 (2.2) | >0.9 |
| TMT-A, time in seconds | 77.4 (41.3) | 41.3 (15.5) | <0.001*** | 66.4 (39.3) | 44.1 (21.1) | <0.05* |
| Gait Speed 6m, m/s | 1.2 (0.2) | 1.3 (0.2) | <0.001*** | 1.2 (0.3) | 1.3 (0.2) | 0.13 |
| Handgrip, kg | 25.0 (7.7) | 29.6 (8.8) | <0.001*** | 30.6 (7.4) | 29.2 (8.8) | 0.4 |
| Peak Leg Muscle Power, W | 173.4 (93.5) | 249.6 (104.5) | <0.001*** | 203.6 (89.3) | 244.0 (105.8) | 0.2 |

*Mean (SD); n (%).*
