# Supplementary material for: Physical Performance Moderates the Association Between Age and Executive Function in Older Adults at Elevated Risk of Dementia: Cross‐Sectional Analysis From the CITA GO‐ON Trial
Source: J Cachexia Sarcopenia Muscle. 2026 Oct 5;17(5):e70396. doi: 10.1002/jcsm.70396 (PMC13638253; doi:10.1002/jcsm.70396)
Supplement: Supplementary file 6 [file JCSM-17-e70396-s006.docx]

Supplementary Table 9. Comparison of participants with versus without MRI-derived WMH data.

| **Characteristic** | **N** | **Excluded**  N = 146 | **Included**  N = 774 | **p-value** |
| --- | --- | --- | --- | --- |
| Age, years | 920 | 68.9 (6.2) | 69.7 (6.2) | 0.12 |
| Sex | 920 |  |  | >0.9 |
| Women |  | 71 (49%) | 374 (48%) |  |
| Men |  | 75 (51%) | 400 (52%) |  |
| Education level, years | 920 | 13.9 (4.5) | 12.8 (4.5) | 0.008 |
| Socioeconomic Status Index | 920 | 2.0 (1.1) | 1.8 (1.1) | 0.15 |
| Syndromic Diagnosis | 920 |  |  | 0.4 |
| Cognitively Healthy |  | 132 (90%) | 674 (87%) |  |
| Subjective Cognitive Decline |  | 8 (5.5%) | 43 (5.6%) |  |
| Mild Cognitive Impairment |  | 6 (4.1%) | 57 (7.4%) |  |
| Other |  | 0 (0%) | 0 (0%) |  |
| Body Mass Index | 917 | 28.2 (5.6) | 27.3 (4.5) | 0.2 |
| GDS score | 919 | 1.7 (1.9) | 2.0 (2.2) | 0.4 |
| TMT-A, time in seconds | 920 | 41.4 (17.4) | 45.0 (22.3) | 0.067 |
| TMT-B, time in seconds | 841 | 99.1 (66.9) | 106.2 (62.0) | 0.054 |
| TMT-B minus TMT-A, time in seconds | 841 | 60.5 (62.1) | 64.4 (54.2) | 0.12 |
| Stroop interference, time in seconds | 907 | 27.7 (20.3) | 26.9 (14.3) | 0.8 |
| Gait Speed 6m, m/s | 898 | 1.3 (0.2) | 1.3 (0.2) | 0.8 |
| Handgrip, kg | 893 | 30.1 (9.3) | 29.1 (8.7) | 0.2 |
| Peak Leg Muscle Power, W | 863 | 248.0 (110.5) | 242.7 (104.9) | 0.7 |

*Mean (SD); n (%).*
