# Supplementary material for: Physical Performance Moderates the Association Between Age and Executive Function in Older Adults at Elevated Risk of Dementia: Cross‐Sectional Analysis From the CITA GO‐ON Trial
Source: J Cachexia Sarcopenia Muscle. 2026 Oct 5;17(5):e70396. doi: 10.1002/jcsm.70396 (PMC13638253; doi:10.1002/jcsm.70396)
Supplement: Supplementary file 7 [file JCSM-17-e70396-s007.docx]

Supplementary Table 8. Comparison of participants with versus without physical performance data.

| **Characteristic** | **N** | **Excluded**  N = 60 | **Included**  N = 860 | **p-value** |
| --- | --- | --- | --- | --- |
| Age, years | 920 | 70.9 (6.9) | 69.5 (6.1) | 0.12 |
| Sex | 920 |  |  | 0.7 |
| Female |  | 31 (52%) | 414 (48%) |  |
| Male |  | 29 (48%) | 446 (52%) |  |
| Education level, years | 920 | 12.4 (4.4) | 13.0 (4.5) | 0.4 |
| Socioeconomic Status Index | 920 | 1.6 (1.2) | 1.9 (1.1) | 0.066 |
| Syndromic Diagnosis | 920 |  |  | 0.12 |
| Cognitively Healthy |  | 49 (82%) | 757 (88%) |  |
| Subjective Cognitive Decline |  | 3 (5.0%) | 48 (5.6%) |  |
| Mild Cognitive Impairment |  | 8 (13%) | 55 (6.4%) |  |
| Body Mass Index | 917 | 28.0 (5.7) | 27.4 (4.6) | 0.7 |
| Fazekas Scale, 0-3 | 774 |  |  | 0.009 |
| 0 |  | 4 (8.7%) | 162 (22%) |  |
| 1 |  | 19 (41%) | 362 (50%) |  |
| 2 |  | 20 (43%) | 177 (24%) |  |
| 3 |  | 3 (6.5%) | 27 (3.7%) |  |
| GDS score | 919 | 2.0 (2.4) | 1.9 (2.1) | 0.8 |
| TMT-A, time in seconds | 920 | 51.9 (25.8) | 43.9 (21.2) | 0.004 |
| TMT-B, time in seconds | 841 | 120.5 (112.5) | 104.1 (58.4) | 0.7 |
| TMT-B minus TMT-A, time in seconds | 841 | 75.6 (103.0) | 63.1 (51.2) | 0.3 |
| Stroop interference, time in seconds | 907 | 32.5 (27.4) | 26.7 (14.2) | 0.10 |

*Mean (SD); n (%).*
