# Supplementary material for: Physical Performance Moderates the Association Between Age and Executive Function in Older Adults at Elevated Risk of Dementia: Cross‐Sectional Analysis From the CITA GO‐ON Trial
Source: J Cachexia Sarcopenia Muscle. 2026 Oct 5;17(5):e70396. doi: 10.1002/jcsm.70396 (PMC13638253; doi:10.1002/jcsm.70396)
Supplement: Supplementary file 8 [file JCSM-17-e70396-s008.docx]

Supplementary Table 4. Moderation (age × performance) additionally adjusted for white-matter hyperintensities (Fazekas).

| **Outcome** | **Physical performance** | **Whole β (95% CI)** | **p** | **Female β (95% CI)** | **p** | **Male β (95% CI)** | **p** |
| --- | --- | --- | --- | --- | --- | --- | --- |
| TMT B minus A | Gait Speed (per 0.1 m/s) | -0.247 (-0.502–0.009) | 0.06 | 0.116 (-0.290–0.522) | 0.57 | -0.521 (-0.863–-0.179) | <0.01** |
|  | Handgrip | -0.069 (-0.144–0.006) | 0.07 | -0.041 (-0.259–0.178) | 0.72 | -0.137 (-0.289–0.016) | 0.08 |
|  | Leg Muscle Power | -0.006 (-0.012–0.000) | 0.05 | -0.005 (-0.023–0.012) | 0.56 | -0.004 (-0.013–0.005) | 0.43 |
| Stroop Interference | Gait Speed (per 0.1 m/s) | -0.056 (-0.122–0.010) | 0.1 | -0.061 (-0.164–0.043) | 0.25 | -0.040 (-0.129–0.049) | 0.38 |
|  | Handgrip | -0.010 (-0.029–0.009) | 0.29 | -0.010 (-0.065–0.044) | 0.71 | -0.003 (-0.042–0.035) | 0.86 |
|  | Leg Muscle Power | -0.001 (-0.002–0.001) | 0.29 | -0.001 (-0.005–0.003) | 0.61 | -0.000 (-0.003–0.002) | 0.75 |

*WMH, white-matter hyperintensities; 95% CI, 95% confidence interval. *p<0.05; **p<0.01; ***p<0.001*
