# Supplementary material for: Physical Performance Moderates the Association Between Age and Executive Function in Older Adults at Elevated Risk of Dementia: Cross‐Sectional Analysis From the CITA GO‐ON Trial
Source: J Cachexia Sarcopenia Muscle. 2026 Oct 5;17(5):e70396. doi: 10.1002/jcsm.70396 (PMC13638253; doi:10.1002/jcsm.70396)
Supplement: Supplementary file 9 [file JCSM-17-e70396-s009.docx]

Supplementary Table 2. Three-way interaction (age × sex × physical performance) on executive function. Adjusted for socioeconomic status, GDS, BMI, anxiety, diabetes, hypertension, dyslipidaemia and smoking.

| **Outcome** | **Physical performance** | **age×sex×marker β (95% CI)** | **p** |
| --- | --- | --- | --- |
| TMT B minus A | Gait Speed | -0.567 (-1.060–-0.073) | **<0.05*** |
|  | Handgrip | -0.133 (-0.360–0.094) | 0.25 |
|  | Leg Muscle Power | 0.000 (-0.016–0.017) | 0.98 |
| Stroop Interference | Gait Speed (per 0.1 m/s) | 0.063 (-0.061–0.187) | 0.32 |
|  | Handgrip | 0.003 (-0.056–0.061) | 0.92 |
|  | Leg Muscle Power | 0.000 (-0.004–0.005) | 0.86 |

*95% CI, 95% confidence interval. *p<0.05; **p<0.01; ***p<0.001*
