# Supplementary material for: Physical Performance Moderates the Association Between Age and Executive Function in Older Adults at Elevated Risk of Dementia: Cross‐Sectional Analysis From the CITA GO‐ON Trial
Source: J Cachexia Sarcopenia Muscle. 2026 Oct 5;17(5):e70396. doi: 10.1002/jcsm.70396 (PMC13638253; doi:10.1002/jcsm.70396)
Supplement: Supplementary file 10 [file JCSM-17-e70396-s010.docx]

**Supplementary Figure 1.** Flow chart of participants and analytic samples.

**Randomized participants**

**N = 920**

**↓**

**Available primary outcome**

TMT-B minus TMT-A

**N = 841**

**Available co-primary outcome**

Stroop interference

**N = 907**

**↓**

**Excluded (N = 79)**

Unable to complete Trail Making Test part B

(alphabetical illiteracy)

**↓**

**Available physical performance measures**

Usual gait speed: N = 898

Handgrip strength: N = 893

Peak leg muscle power: N = 863

**↓**

**Excluded**

Gait N = 22; Handgrip N = 27; Power N = 57

(missing; absolute/relative 1RM contraindication;

unwillingness; staff shortage; unreachable)

**↓**

**Analytic samples**

| **Analytic N** | **Gait speed** | **Handgrip** | **Leg muscle power** |
| --- | --- | --- | --- |
| **TMT B minus A** | 817 | 811 | 787 |
| **Stroop interference** | 877 | 872 | 846 |

Note. Analytic N values correspond to the complete-case sample available for each age × physical-performance interaction model after listwise deletion of missing outcome, predictor, moderator, and covariate data (socioeconomic status, depressive symptoms, body-mass index, sex, anxiety, diabetes, hypertension, dyslipidaemia, and smoking).
